# Psychosocial and Behavior Correlates of Early Pornography Exposure in Children

**DOI:** 10.21203/rs.3.rs-9881429/v1

**Published:** 2026-06-23

**Authors:** Kimball Moffat, Jeffrey Glenn, Erik J. Nelson

**Affiliations:** Brigham Young University; Brigham Young University; Brigham Young University

## Abstract

**Background.:**

The early and often unintentional exposure of children to pornographic material has raised growing developmental and public health concerns, particularly given the increasing prevalence of unsupervised digital access.

**Methods.:**

We used baseline data from the Adolescent Brain Cognitive Development (ABCD) Study to examine psychological, behavioral, and demographic correlates of pornography use among 11,455 children aged 9–10 years. Children were classified as exposed to pornography based on parent-reported access within the past two weeks. Pornography exposure was assessed via parent report.

**Results.:**

Compared to the parent-reported non-exposure group, parent-reported exposed children were significantly more likely to have screen time above the recommended amount by the American Academy of Pediatrics Recommendations. Children exposed were significantly more likely to be male, Black, and from lower-income households. Exposed children also exhibited higher rates of diagnosed mental health conditions, including ADHD, anxiety, and depression, as well as elevated screen time, disobedience, and perfectionism.

**Discussion.:**

These findings suggest early pornography exposure may be associated with increased screen time usage and both internalizing and externalizing risk patterns during a critical stage of cognitive and emotional development. The observed disparities by screen time, race, gender, and income highlight the role of broader structural and environmental factors in shaping risk. These results underscore the importance of content-specific digital regulation, parental monitoring, and developmentally tailored interventions. Future longitudinal research is needed to assess long-term outcomes and evaluate the potential protective impact of early clinical or behavioral interventions.

## BACKGROUND

The rapid expansion of internet access has fundamentally reshaped how individuals, including children, engage with digital content.^1^ While this technological advancement offers educational and recreational benefits, prior research has identified early and often unintentional exposure of children as young as eight years old to pornographic material as a potential concern.^2^ Such early exposure is especially considered concerning in the literature, given the critical developmental window marked by cognitive, emotional, and social changes that shape identity and worldview.^2^ In this study, we focus specifically on preadolescent children aged 9–10 years.

During these early stages, children are forming foundational understandings of relationships, gender roles, and sexuality. Exposure to pornographic content during this period has been associated with unrealistic expectations, normalization of objectification, and acceptance of harmful relationship dynamics.^2^ These effects are often experienced differently by individuals of different genders. Boys are more likely to encounter and actively engage with pornography, and they often perceive it more positively.^3^ In contrast, girls are more likely to report discomfort, confusion, or duress when exposed, which has been associated with lower self-esteem and body image concerns.^4^ Gender-specific responses to exposure may be associated with differences in the development of sexual attitudes, interpersonal behaviors, and emotional well-being in distinctive ways.

Beyond gender, previous research has found that some groups are more likely to experience pornography exposure at a young age. Early exposure is facilitated by immediate and autonomous internet access, with pornography often viewed privately or in groups in largely unsupervised settings.^5^ Racial and ethnic patterns have also been noted, with urban low-income Black and Hispanic individuals reporting easy access, frequent viewing in peer settings, and preferences for same-race actors.^6^

The psychological impact of pornography exposure is further influenced by factors such as gender, family environment, and cultural and religious values. Although greater religiosity has been linked to lower pornography use, children raised in settings with strong ethical or faith-based teachings may report greater feelings of guilt, shame, or confusion following exposure to pornography, which can be particularly damaging during this formative stage.^7^ Similar challenges have also been observed in nonreligious contexts, where factors such as low self-esteem, family conflict, or limited parental monitoring can shape distressing patterns of use. Additionally, problematic use patterns among these children may emerge – more frequently among boys – characterized by impulsivity, secrecy, and sexual distress, all of which have been associated with difficulties in emotional regulation and social development.^5^

One key pathway through which these structural and psychosocial factors may influence exposure risk is through children’s overall screen time. Previous research suggests increased screen exposure is associated with greater accessibility to explicit content.^5^ Emerging evidence also reports associations between heavier screen exposure in childhood and both desensitization and poorer mental health. Longitudinal data suggest that high screen time is associated with blunted reward responsiveness through anhedonia, a pathway implicated in greater reward-seeking risk behavior.^8^ Meta-analytic and review evidence further indicates small but consistent associations between greater screen time and externalizing and internalizing problems in children, with the strongest and most consistent findings for depressive symptoms and lower quality of life.^9, 10^ These externalization and internalization of problems may be linked to disobedience and perfectionism, respectively. Because ages 9–10 are a key period for developing self-control and coping strategies, these behavioral patterns may help identify children who are vulnerable to distressing or problematic digital experiences.^11^ Patterns motivate our focus on background screen exposure as a contextual risk factor associated with early exposure to sexual content.

There remains some disagreement among public health experts and elected officials regarding the prioritization of pornography exposure as a public health issue. While multiple states have passed legislation declaring pornography a public health crisis, some researchers argue that focusing scarce resources on the issues takes away from more pressing problems.^12^ There is a growing body of evidence of the harms of pornography exposure to merit a public health response. Still, the continued debate underscores the importance of further research to clarify its scope and guide policy.^13, 14^

Despite growing awareness, there remains a critical gap in research focused on the correlates of pornography exposure on young children. This study sought to address that gap by examining key developmental, psychological, and behavioral risks associated with early exposure to pornography and identifying demographic patterns that contribute to vulnerability. Gaining a deeper understanding of these dynamics is essential for developing effective interventions and informing policies that protect all children during this pivotal stage of development. Because much of the existing literature is observational, associations described here should not be interpreted as causal.

## METHODS

This study was granted exempt status from the Brigham Young University IRB Office.

### Study Participants

Participants were selected from the Adolescent Brain Cognitive Development (ABCD) study, the largest single-cohort, longitudinal study of neurodevelopment and children’s health in the United States. The ABCD study recruited children aged 9–10 years old and their parents from 21 study sites across the U.S., beginning in 2018. Sampling was done through elementary schools, ensuring a representative sample of national demographics in terms of gender, race, ethnicity, socioeconomic status, and urbanicity.^15^ For this study, we used baseline data from the ABCD 5.0 data release, which included 11,862 children aged 9–10 years old. Participants were classified as exposed to pornography if their parents reported that their child had downloaded or accessed pornography within the past two weeks. Participants with missing data on pornography use (n = 242) and race/ethnicity (n = 2) were excluded, resulting in a final analytic sample size of 11,618 children.

### Measures

#### Pornography Use

Pornography use was determined based on parental responses to the question: “Has your child downloaded or accessed pornography in the past two weeks?” Children whose parents responded “yes” were classified as exposed to pornography, while those whose parents responded “no” were categorized as not exposed to pornography.

#### Disobedience and Perfectionism

To assess behavioral tendencies, parents responded to two separate questions about their child’s behavioral traits: “Is your child disobedient at home?” and “Does your child feel the need to be perfect?” Children whose parents answered “no” to the first question and “yes” to the second question were classified as exhibiting higher levels of obedience or perfectionism, respectively.

#### Screen Time

Screen time was assessed through parent-reported measures of weekly and weekend screen use. Parents were asked to estimate the number of hours their child spent on screens (e.g., television, computers, tablets, and smartphones) during an average weekday and weekend day. We multiplied weekday responses by 5 to represent the average weekday hours viewed, as well as weekend days by a factor of 2, then summed all screen time hours to estimate the average weekly screen time for each child. This resulted in an average of 2 hours per day for 10 hours total during weekdays, and an average of 2 hours per day on weekends for a total of 4 hours. In all, the recommended screen time in a given week for 9–10-year-old children is 14 hours. The total estimated weekly screen time was then categorized as compliant or not with the American Academy of Pediatrics (AAP) recommendations for screen time^16^ with the cut point of 14 total viewing hours per week.

#### Mental Health

Mental health status was evaluated using parental reports of diagnosed mental health conditions, including attention-deficit/hyperactivity disorder (ADHD), depression, and anxiety disorders. Parents were asked whether their child had ever received a clinical diagnosis of any of these conditions (yes/no).

#### Demographics

Biological sex was determined based on the parental reports of the child’s assigned sex at birth (male or female). Ethnicity was recorded based on parental identification of the child’s race/ethnic background (White, Black, Hispanic, Asian, or other). Household income was reported in the following categories: $0–$11,999; $12,000–$24,999; $25,000–$49,999; $50,000–$99,999; $100,000–$199,999; and $200,000 or more.

#### Statistical Analysis

We tabulated the frequency of the aforementioned variables by pornography use. We used chi-square tests (or Fisher’s exact tests where appropriate) to assess differences between those exposed and those not exposed to pornography. All analyses and data visualizations were conducted in R version 4.2.1.

## RESULTS

Table 1 presents the demographic characteristics of the 11,618 children aged 9 to 10 years old included in the sample. Of these, 152 (1.3%) were classified as exposed to pornography. A notable gender disparity was observed, with males comprising 70.4% of those exposed, compared to 29.6% who were female.

Differences by race and ethnicity were also evident. Black (27.6%) children were overrepresented among those exposed to pornography relative to their proportions in the non-exposed group (14.9%). White (35.5%) and Asian (1.3%) children comprised a smaller share of those exposed compared to non-exposed (52.4% vs. 2.1%, respectively).

Characteristics of screen time by pornography use are presented in Table 2. Children who were reported as viewing pornography reported significantly higher average total weekly screen time, with those exposed to pornography spending 23 hours on screens per week compared to 16 hours among those not exposed. This translates to 74% of those exposed and 56% of non-exposed who have screen time use beyond the AAP recommendations. A similar pattern of higher screen time emerged for both weekday screen time and weekend screen time, with those exposed to pornography consistently reporting longer durations.

As shown in Table 3, mental health conditions were more prevalent among those exposed to pornography, with 24.3% reporting a diagnosis, compared to 14.5% of those not exposed. Behavioral traits also differed markedly. Disobedience was reported in 69.7% of those exposed compared to 38.7% of the non-exposed. Likewise, perfectionist tendencies were noted in 36.8% of those exposed, compared to 27.1% of those non-exposed.

In summary, parent-reported pornography use among children was associated with several distinct characteristics. Those exposed were more likely to be male and from Black or Hispanic backgrounds, had significantly higher screen time on both weekdays and weekends, and were more likely to have mental health diagnoses. They also showed elevated rates of disobedience and perfectionism.

## DISCUSSION

Using baseline data from the Adolescent Brain Cognitive Development (ABCD) Study, we found that children aged 9–10 who were parent-reported to have viewed pornography within the past two weeks were disproportionately male, were roughly equally spread across household income groups, and had higher proportions of those exposed than those not exposed among Black children. Compared to the non-exposed, children who had viewed pornography showed significantly higher rates of disobedient behavior, perfectionism, greater screen time, and diagnosed mental health conditions. These patterns are concerning, and given the developmental sensitivity of this age group, they reinforce prior research suggesting that early exposure to sexually explicit material may be linked to emotional dysregulation, behavioral difficulties, and maladaptive coping mechanisms. The overrepresentation of racial and ethnic minority children in our results, particularly those from lower-income households, aligns with prior ABCDbased findings showing how structural and environmental inequities contribute to increased vulnerability to mental health and behavioral challenges during middle childhood.^6^ In this context, pornography exposure may serve as a marker of broader risk patterns related to unmonitored and unregulated digital access and underlying systemic disadvantages.

Our data also showed that a higher proportion of parent-reported pornography exposed individuals, compared to the non-exposed individuals, exceeded the American Academy of Pediatrics’ recommendations for both weekday and weekend screen time. Excessive screen time has been shown to blunt reward sensitivity via anhedonia, which in turn is associated with increased engagement in risky behaviors.^8^ Heavy, low-quality digital use is further linked to emotional desensitization and cognitive overload.^17^ Evidence consistently shows that children under 12 who spend more time on screens are at greater risk for both externalizing and internalizing problems.^9^ Although our cross-sectional design prevents causal conclusions, the co-occurrence of elevated screen time and recent pornography exposure in 9–10-year-old children is consistent with these mechanisms.

Our data revealed a strong association between parent-reported pornography use and increased screen time, suggesting a potential dose-response relationship in which greater digital exposure corresponds with higher engagement in risk behaviors,^18^ including access to explicit content. This aligns with prior findings that heavy, unsupervised computer use–more than television or video games–is linked to significantly elevated risk-taking behaviors.^18^ Such patterns may reflect both greater access to unfiltered online content and reduced parental oversight. For adolescents of color, greater screen time has also been tied to increased exposure to online racial discrimination and related mental health burdens, underscoring how digital engagement can reinforce cycles of stress and maladaptive coping.^19^

Importantly, these findings highlight that screen time is not content-neutral; both the type and duration of exposure matter. In today’s digital environment-where children are frequently exposed to sexual content, cyber-victimization, and misinformation-effective interventions must extend beyond limiting screen time to include content filtering, enhanced parental monitoring, and developmentally appropriate digital literacy education programs.

The elevated prevalence of ADHD, anxiety, and depression among those exposed to pornography supports the mood management hypothesis, which suggests that individuals may use pornography to self-regulate negative emotions or alleviate psychological distress. The ADHD association is consistent with prior evidence that ADHD symptoms are linked to problematic internet use and related impulsive/compulsive digital engagement, which can increase exposure to high-stimulation content online.^20^ Excessive screen use in youth is linked to poorer mental health, lower quality of life,^10^ and emotional desensitization.^21^ Within this context, our finding that pornography-exposed children had higher rates of mental health diagnoses aligns with evidence that psychological distress is associated with early exposure to explicit content. Recent studies also show depressive symptoms are significantly associated with more frequent pornography use, particularly among boys, with odds ratios as high as 2.72 for those characterized as exposed.^22^ Because children at this age are still developing executive functioning and emotional regulation, traits such as perfectionism (an internalizing symptom) and disobedience (an externalizing behavior) may further increase risk for compulsive digital engagement. These findings highlight the need for gender-sensitive, trauma-informed, and developmentally tailored prevention strategies that extend beyond content blocking.

While pornography exposure may not be universally recognized as a public health crisis, our findings that specific adolescent vulnerabilities are associated with exposure suggest the need to use public health tools to protect adolescents from its potentially harmful effects at this critical developmental stage. Common-sense policies such as enhancing parental monitoring, strengthening school-based digital literacy education, and expanding access to developmentally appropriate resources should be developed and rigorously evaluated. In this way, prevention efforts can move beyond individual responsibility to address broader structural and environmental factors that contribute to early and unmonitored exposure.

Utah’s 2023 legislation provides an early example of state-level efforts to restrict minors’ access to sexually explicit content by requiring commercial websites to verify viewer age through digitized identification, third-party services, or comparable methods.^23^ As of 2025, 24 states- including Louisiana, Texas, Mississippi, and Virginia- have enacted similar laws. Legal challenges^24^ show concern about privacy and legal validity, yet these policies show an approach where laws and regulations work alongside parental guidance and digital literacy programs to help reduce risks of early exposure to pornography.

This study has several important limitations. First, the cross-sectional nature of the data prevents any conclusions about causality. While significant associations were observed between pornography use and various psychosocial and behavioral outcomes, the directionality of these relationships cannot be determined. Second, all measures relied on parent report, which may be subject to reporting bias. It is suspected that parents may underreport behaviors they perceive as stigmatized or may be unaware of their child’s private digital activity, leading to potential misclassification of pornography exposure or related traits. Findings may reflect parental perceptions of children’s behavior rather than children’s actual behavior. Accordingly, results should be interpreted as associations between parent-report exposure to pornography and psychosocial characteristics. Third, although the ABCD Study is designed to provide a demographically representative sample of U.S. children, it may not fully capture the diversity of experiences across all cultural, geographic, and socioeconomic contexts in the U.S. Fourth, pornography exposure was assessed using a parent-reported item, lacking a standardized definition of “pornography” or information on content type, platform, frequency, or duration. Exposure may reflect a variety of materials, limiting comparability with other studies that may have a more specific measure of what is “pornography.”

We found that children who engaged with pornography at ages 9–10 were more likely to exhibit behavioral challenges, mental health diagnoses, and have elevated screen time, with notable disparities by gender and race. Future longitudinal research should examine whether early exposure to pornography compounds psychological and behavioral risks over time and whether early behavioral or clinical interventions can buffer against long-term emotional and cognitive consequences.

## Figures and Tables

**Table 1:** Sex, Race/Ethnicity and Household Income Differences of Children by Pornography Exposure Status

Characteristic	Pornography Exposure			p-value^2^
	Overall N = 11,618^1^	Yes N = 152^1^	No N = 11,466^1^	
**Sex at Birth**				**< 0.001**
Female	5,537 (47.7%)	45 (29.6%)	5,492 (47.9%)	
Male	6,081 (52.3%)	107 (70.4%)	5,974 (52.1%)	
**Race/Ethnicity**				**< 0.001**
White	6,064 (52.2%)	54 (35.5%)	6,010 (52.4%)	
Hispanic	2,336 (20.1%)	33 (21.7%)	2,303 (20.1%)	
Black	1,747 (15.0%)	42 (27.6%)	1,705 (14.9%)	
Asian	244 (2.1%)	2 (1.3%)	242 (2.1%)	
Other	1,227 (10.6%)	21 (13.8%)	1,206 (10.5%)	
**Household Income**				**< 0.001**
<$24,999	1,597 (13.7%)	33 (21.7%)	1,564 (13.6%)	
$25,000–$49,999	1,550 (13.3%)	43 (28.3%)	1,507 (13.1%)	
$50,000–$99,999	2,999 (25.8%)	37 (24.3%)	2,962 (25.8%)	
$100,000–$199,999	3,247 (27.9%)	27 (17.8%)	3,220 (28.1%)	
>$200,000	1,232 (10.6%)	4 (2.6%)	1,228 (10.7%)	
Missing	993 (8.5%)	8 (5.3%)	985 (8.6%)	

1 n (%)

2 Pearson’s Chi-squared test; Fisher’s exact test

**Table 2:** Mental Health and Behavioral Traits of Children by Pornography Use Status

Characteristic	Pornography Use			p-value^2^
	Overall N = 11,581^1^	Yes N = 151^1^	No N = 11,430^1^	
**Mental Health Conditions**				**< 0.001**
Yes	1,703 (14.7%)	37 (24.5%)	1,666 (14.6%)	
No	9,878 (85.3%)	114 (75.5%)	9,764 (85.4%)	
**Feels the Need to Be Perfect**				**0.006**
Yes	3,159 (27.3%)	56 (37.1%)	3,103 (27.1%)	
No	8,422 (72.7%)	95 (62.9%)	8,327 (72.9%)	
**Disobedient**				**< 0.001**
Yes	4,537 (39.2%)	105 (69.5%)	4,432 (38.8%)	
No	7,044 (60.8%)	46 (30.5%)	6,998 (61.2%)	

1 n (%)

2 Pearson’s Chi-squared test

**Table 3:** Screen time Use of Children by Pornography Use Status

Characteristic	Pornography Use		
	Yes N = 152^1^	No N = 11,458^1^	p-value^2^
**Screen Time Hours by American Academy of Pediatrics Recommendations**			
**Total Screen Time**			**< 0.001**
<14 hours	39 (26%)	5,086 (44%)	
>14 hours	113 (74%)	6,372 (56%)	
**Weekday Screen Time**			**< 0.001**
<10 hours	37 (24%)	4,711 (41%)	
>10 hours	115 (76%)	6,747 (59%)	
**Weekend Screen Time**			**0.033**
<4 hours	11 (7.2%)	1,499 (13%)	
>4 hours	141 (93%)	9,959 (87%)	
**Average Screen Time Hours**			
**Total Screen Time (Mean)**	23 (14, 30)	16 (9, 25)	**< 0.001**
**Weekday Screen Time (Mean)**	15 (10, 20)	10 (5, 15)	**< 0.001**
**Weekend Screen Time (Mean)**	8.0 (6.0, 12.0)	6.0 (4.0, 10.0)	**< 0.001**

1 n (%); Median (Q1, Q3)

2 Pearson’s Chi-squared test; Wilcoxon rank sum test

## Data Availability

Data used in the preparation of this article were obtained from the Adolescent Brain Cognitive DevelopmentSM (ABCD) Study (https://abcdstudy.org), held in the NIMH Data Archive (NDA). This is a multisite, longitudinal study designed to recruit more than 10,000 children age 9–10 and follow them over 10 years into early adulthood. The ABCD Study^®^ is supported by the National Institutes of Health and additional federal partners under award numbers U01DA041048, U01DA050989, U01DA051016, U01DA041022, U01DA051018, U01DA051037, U01DA050987, U01DA041174, U01DA041106, U01DA041117, U01DA041028, U01DA041134, U01DA050988, U01DA051039, U01DA041156, U01DA041025, U01DA041120, U01DA051038, U01DA041148, U01DA041093, U01DA041089, U24DA041123, U24DA041147. A full list of supporters is available at https://abcdstudy.org/federal-partners.html. A listing of participating sites and a complete listing of the study investigators can be found at https://abcdstudy.org/consortium_members/. ABCD consortium investigators designed and implemented the study and/or provided data but did not necessarily participate in the analysis or writing of this report. This manuscript reflects the views of the authors and may not reflect the opinions or views of the NIH or ABCD consortium investigators. Additional support for this work was made possible from NIEHS R01-ES032295 and R01-ES031074.

## What’s Known on This Subject

Early pornography exposure has been linked to negative developmental, behavioral, and emotional outcomes in adolescents, but little is known about its psychosocial and demographic correlates among younger children aged 9–10 in large, nationally representative samples.

### What This Study Adds:

This study identifies significant links between early pornography exposure and increased screen time, behavioral issues, and mental health diagnoses in children aged 9–10, highlighting disparities and emphasizing the need for early, targeted prevention efforts.

## Abbreviations:

ABCD: Adolescent Brain Cognitive Development
